# Postnatal Conditional Deletion of *Bcl11b* in Striatal Projection Neurons Mimics the Transcriptional Signature of Huntington’s Disease

**DOI:** 10.3390/biomedicines10102377

**Published:** 2022-09-23

**Authors:** Sicheng Song, Jordi Creus Muncunill, Carlos Galicia Aguirre, Kizito-Tshitoko Tshilenge, B. Wade Hamilton, Akos A. Gerencser, Houda Benlhabib, Maria-Daniela Cirnaru, Mark Leid, Sean D. Mooney, Lisa M. Ellerby, Michelle E. Ehrlich

**Affiliations:** 1 Department of Biomedical Informatics and Medical Education, School of Medicine, University of Washington, Seattle, WA 98109, USA; 2 Department of Neurology, Icahn School of Medicine at Mount Sinai, New York, NY 10029, USA; 3 Buck Institute for Research on Aging, Novato, CA 94945, USA; 4 Leonard Davis School of Gerontology, University of Southern California, 3715 McClintock Ave, Los Angeles, CA 90893, USA; 5 Department of Pharmaceutical Sciences, College of Pharmacy and Pharmaceutical Sciences, Washington State University, Spokane, WA 99202, USA

**Keywords:** BCL11B, CTIP2, Huntington’s disease, striatal medium spiny neurons, transcriptomics, induced pluripotent stem cells, Bcl11b conditional knockout

## Abstract

The dysregulation of striatal gene expression and function is linked to multiple diseases, including Huntington’s disease (HD), Parkinson’s disease, X-linked dystonia-parkinsonism (XDP), addiction, autism, and schizophrenia. Striatal medium spiny neurons (MSNs) make up 90% of the neurons in the striatum and are critical to motor control. The transcription factor, *Bcl11b* (also known as *Ctip2*), is required for striatal development, but the function of *Bcl11b* in adult MSNs in vivo has not been investigated. We conditionally deleted *Bcl11b* specifically in postnatal MSNs and performed a transcriptomic and behavioral analysis on these mice. Multiple enrichment analyses showed that the *D9-Cre-Bcl11b^tm1.1Leid^* transcriptional profile was similar to the HD gene expression in mouse and human data sets. A Gene Ontology enrichment analysis linked *D9-Cre-Bcl11b^tm1.1Leid^* to calcium, synapse organization, specifically including the dopaminergic synapse, protein dephosphorylation, and HDAC-signaling, commonly dysregulated pathways in HD. *D9-Cre-Bcl11b^tm1.1Leid^* mice had decreased DARPP-32/*Ppp1r1b* in MSNs and behavioral deficits, demonstrating the dysregulation of a subtype of the dopamine D2 receptor expressing MSNs. Finally, in human HD isogenic MSNs, the mislocalization of BCL11B into nuclear aggregates points to a mechanism for BCL11B loss of function in HD. Our results suggest that BCL11B is important for the function and maintenance of mature MSNs and *Bcl11b* loss of function drives, in part, the transcriptomic and functional changes in HD.

## 1. Introduction

The basal ganglia comprise interconnected subcortical nuclei that are responsible for motor learning and control, executive functions, and emotions. The striatum, composed of the caudate and putamen in humans, is the largest component of the basal ganglia. It receives and integrates glutamatergic and dopaminergic inputs from several brain regions, including the cortex, thalamus, hippocampus, and amygdala. These inputs target inhibitory γ-amino butyric acid (GABA)-ergic medium spiny neurons (MSNs), the principal output neurons of the striatum, and make up 90–95% of its total neurons. MSNs are morphologically homogeneous; however, they can be distinguished by their output targets and their specific gene expression. Direct MSNs project to the global pallidus internal or substantia nigra pars reticula and express the dopamine D1 receptor (*Drd1*), substance P, and dynorphin. Indirect MSNs project to the globus pallidus external and express the dopamine D2 receptor (*Drd2*), the adenosine 2A receptor, and enkephalin.

The dysfunction and developmental alterations of MSNs or their subtypes have been implicated in several neurological and neuropsychiatric disorders, including Huntington’s disease (HD) [1], dystonia [2] (e.g., X-linked dystonia parkinsonism [3,4]), addiction [5,6], and schizophrenia [7]. Some of these diseases manifest in late adulthood and include prominent gene transcriptional abnormalities in mature MSNs. Disrupted levels and/or the activity of specific transcription factors (TFs) involved in development were described as a potential common pathogenic mechanism underlying cell type-specific vulnerability [8,9]. TFs play a key role in controlling the spatiotemporal expression of cell type-specific genes. For this reason, knowledge of TF activity in mature MSNs is crucial to understanding striatal-related pathologies. One critical TF involved in the striatal development of MSNs is B-cell leukemia 11b, *Bcl11b* (also known as *Ctip2*).

CTIP1 and CTIP2 were first identified in mouse neurons as Krüppel-like C(2)H(2) zinc finger proteins interacting with all members of the chicken ovalbumin upstream promoter TF family (COUP-TF) [10]. CTIP1, later named BCL11A, is highly enriched in COUP-TF-negative cells of the immune system and it is also expressed in the cerebral cortex [11,12,13]. *CTIP2*, located on chromosome 14, is the homologue of CTIP1 and was named *BCL11B* [14]. Both are sequence-specific DNA-binding proteins that repress or induce transcription [10]. *Bcl11b* is highly expressed in the embryonic cortex and striatum. Interestingly, unlike most developmental striatal TFs, *Bcl11b* levels remain high throughout life [15,16,17,18]. Therefore, exploration of the role of *Bcl11b* in mature MSNs is essential. Importantly, BCL11B activity is dysregulated in HD patients and models, suggesting that it participates in the maintenance and function of mature MSNs [1,17,19,20,21,22].

*Bcl11b* is required for the development of corticospinal motor neuron projections and the differentiation of MSNs [10,23,24], and is frequently used as a pan-MSN marker, along with DARPP-32/*Ppp1r1b*, which is expressed later in development relative to *Bcl11b* [16,20,25,26,27]. Several in vitro and vivo *Bcl11b*-null models have been characterized. Constitutive murine *Bcl11b* deletion results in altered striatal compartmentalization as determined by the absence of DARPP-32 in the late embryonic striatum and in the aberrant expression of genes associated with both direct and indirect pathways [15]. The loss of function and Bcl11b ChIP-sequencing experiments in an immortalized striatal cell line indicated that *Bcl11b* regulates the expression of striatal-enriched genes [17,28] and the genes involved in the BDNF signaling pathway [28]. In MSNs derived from human embryonic stem cells, BCL11B regulates the expression of genes related to Ca^2+^ signaling and kinase activity, playing a crucial role in MSN homeostasis [20]. A consensus motif for Bcl11b has been proposed [10,28], but the binding site is considered “promiscuous”, and Bcl11b is also part of nucleosome remodeling and deacetylation complexes, NuRD and SWI/SNF, suggesting that it regulates gene expression through mechanisms that are not restricted to direct DNA binding. Notably, the functions of *Bcl11b* specific to adult MSNs have not been elucidated and are the focus of our current study.

Much is known about TFs and their development, but little is known, particularly in neuronal subtypes, about TFs and neuronal phenotype maintenance, which is an active process [29]. To determine the role of *Bcl11b* in adult MSNs in vivo, the regulatory components of the genomic elements of DARPP-32 (i.e., D9) [30] were used to delete the expression of *Bcl11b,* selectively, in post-mitotic, post-migrational MSNs (*D9-Cre-Bcl11b^tm1.1Leid^*). We provide evidence that *Bcl11b* deletion results in a decrease in the expression of the genes involved in general neuronal survival and maintenance, and a decrease in the markers that characterize the unique MSN subtypes. Furthermore, we found that specific dopamine receptor-mediated behavior is impacted. Compellingly, the transcriptomic profile of *D9-Cre-Bcl11b^tm1.1Leid^* mice significantly overlaps with the gene expression changes in HD human and mouse striatum. A Gene Ontology (GO) enrichment analysis links *Bcl11b* deletion to calcium and HDAC signaling pathways that are commonly dysregulated in HD, and to MSN-specific pathways, which are also dysregulated in HD. Finally, in human HD-isogenic MSNs, the mislocalization of BCL11B into nuclear aggregates points to a mechanism for BCL11b loss of function in HD.

## 2. Materials and Methods

**Mice:** Experimental procedures were carried out according to the Institutional Animal and Care and Use Committee at Icahn School of Medicine at Mount Sinai (LA09-00272, 16-0847 PRYR1). We followed the NIH Guidelines for the Care and Use of Experimental Animals. Bcl11b-floxed mice were obtained from Dr. Mark Leid (*Bcl11b^tm1.1Leid^/J*, #034469, Jackson Laboratory, West Grove, PA, USA). D9-Cre mice were created in our laboratory [30]. Cre expression was controlled by a regulatory element of the mouse Ppp1r1b gene-encoding DARPP-32. The breeding of these lines resulted in a *Bcl11b*-specific deletion in MSNs at 5–6 weeks of age. Both females and males were used. Food and water was provided ad libitum and mice were kept in a 12-h light-dark cycle.

**Tissue extraction:** Pentobarbital (50 mg/kg) was intraperitoneally injected into mice followed by perfusion with phosphate-buffered saline (PBS, Fisher Bioreagents, Pittsburgh, PA, USA, BP399-1, 4 °C). The hemispheres of the brains were sagittally separated. The left hemisphere striatum was flash frozen. The frozen striatum was used for RNA extraction. The right hemisphere was fixed in 4% paraformaldehyde (Electron Microscopy Sciences, 15710).

**Immunofluorescence and image acquisition:** Brains were sectioned coronally on a vibratome (Leica Microsystems, Wetzlar, Germany) at 30 µm. Brain sections were washed with 1X Tris Buffered Saline (TBS, Fisher Bioreagents; BP2471-1) and incubated in 5% goat serum (ThermoFisher Scientific, Waltham, MA, USA, 31872) with 0.25% Triton X-100 (Sigma Aldrich, St. Louis, MO, USA, X100-500 mL) in TBS for 1 h at room temperature, and then incubated overnight at 4 °C with primary antibodies, as follows: mouse anti-DARPP-32 (1:1000, Santa Cruz Biotechnology, Inc., Santa Cruz, CA, USA, sc-271111), rabbit anti-BCL11B (1:1000, affinity purified, Bethyl Laboratories, Montgomery, TX, USA; A300-385A), rabbit anti-Iba1 (1:500; WAKO Chemicals, Richmond, VA, USA, 019-19741), and rabbit anti-NeuN (1:1000; Millipore; St. Louis, MO, USA, ABN78). Next, sections were incubated with the appropriate secondary antibody: anti-rabbit Alexa 594 (1:400, ThermoFisher Scientific, A-11012, ThermoFisher Scientific), or anti-mouse Alexa 488 (1:400, ThermoFisher Scientific, A-11008). Images were acquired using a Zeiss 700 confocal microscope (Zeiss, Thornwood, NY, USA). For colocalization experiments, we acquired four image frames of three independent brain slices per mouse. Images were also obtained using an Olympus BX61 microscope and processed using Fiji software (ImageJ v1.51).

**RNA-seq:** Dissected striatum from 4-month-old mice were subjected to RNA extraction. The TruSeq RNA Sample Prep Kit v2 protocol (Ilumina, San Diego, CA, USA) was used and the rRNA-depleted libraries were sequenced on the Illumina HiSeq 2500 System with 100 nucleotide paired-end reads. Bases with a quality score lower than 20 and adapted sequences of the raw reads from the sequencing experiment were removed using Trim Galore! 0.6.4. (https://www.bioinformatics.babraham.ac.uk/projects/trim_galore/ (January 2022)). The analysis was performed, as described in [31]. The deposited raw data for the transcriptomics are GSE185476. Differential expression analysis used an arbitrary cutoff of adjusted *p*-value of less than 0.01.

**Terminology enrichment analysis and pathway enrichment analysis:** These were conducted using clusterProfiler, a Bioconductor package in our analysis. All terms in biological process (BP), molecular function, and cellular component categories in GO, as well as pathway annotations derived from Kyoto Encyclopedia of Genes and Genomes (KEGG), were chosen to identify statistically over-represented, biologically meaningful annotations that were enriched and involved in the deletion of *Bcl11b*. We conducted both analyses on the differentially expressed gene and TF clusters with the arbitrary cutoff of the adjusted *p*-value of less than 0.01 and the absolute Log2 fold-change of greater than 0. Data were analyzed using QIAGEN Ingenuity Pathway Analysis.

**Enrichr analysis:** We analyzed for the enrichment of TFs, co-expressors, neural tissues, and GEO sets using Enrichr, an online tool for performing enrichment analysis with a range of biologically meaningful gene-set libraries. In our analysis, the TF perturbation categories in the GEO, ChEA, and ENCODE databases were chosen to identify the significant upstream TFs of those genes differentially expressed in *Bcl11b^tm1.1Leid^* mouse MSNs. The ARCHS4 database was chosen to identify significant co-expressors of those differentially expressed genes and TFs. The Mouse Gene Atlas database was chosen to conduct the neural tissue enrichment analysis to determine the similarity between our mouse *D9-Cre-Bcl11b^tm1.1Leid^* MSNs and other typical neural tissues. Gene sets extracted from perturbations of single genes, drugs, and diseases and RNA-seq disease gene and drug signature categories in the GEO database were chosen to identify synergistic genes of *Bcl11b* and to determine the similarities and differences in our RNA-seq data and high-throughput sequencing data from other peer-reviewed publications. An arbitrary cutoff of an adjusted *p*-value of less than 0.01 was chosen to determine the differentially expressed genes.

**GeneMANIA gene regulatory network analysis:** We used GeneMANIA to conduct the interaction network inference analysis of TFs that were enriched in *D9-Cre-Bcl11b^tm1.1Leid^* MSNs, with the arbitrary cutoff of an adjusted *p*-value of less than 0.01.

**Statistical analysis of transcriptomics:** Fisher’s exact tests were used to determine the overlap of the gene expression profile of the Q175 knockin mouse model [21] and the conditional *D9-Cre-Bcl11b^tm1.1Leid^*. The RNA-seq dataset of 10-month Q175 knockin mouse striatum (GSE 65774) had 2795 differentially expressed genes, and the *D9-Cre-Bcl11b^tm1.1Leid^* had 2771 differentially expressed genes. We used 0.01 as the arbitrarily adjusted *p*-value (FDR) cutoff. The background gene number was 24,106, according to the miRNA and coding gene data from the UCSC mouse mm10 GRCm38 genome assembly. Notably, instead of comparing the overlap of the gene name, we designed a method to compare the overlap of the gene signature, using the Fisher’s exact test twice. For the first test, genes were counted as overlapping genes only if they were differentially expressed in both datasets and shared the same pattern of expression (either upregulated or downregulated) and were in direct correlation between datasets. For the second test, only the genes that were differentially expressed in both datasets and their patterns of expression that were in inverse correlation in both datasets were counted as overlapping genes. Fisher’s test was not statistically significant. Cell-type enrichment analysis was calculated using Chi-square test. Cell-type specific genes were obtained from [32].

### Behavioral Testing of Bcl11b-Deletion Mice

**Locomotor activity:** Spontaneous locomotor activity was measured using the Digiscan D-Micropro automated activity monitoring system (Accuscan, Inc., Columbus, OH, USA). This system consists of transparent plastic boxes (45 × 20 × 20 inch) set inside metal frames that are equipped with 16 infrared light emitters and detectors with 16 parallel infrared photocell beams. Breaks were recorded using a computer interface in 5-min bins. Mice were habituated to the testing chamber for 2 days, and on the third day, their locomotor activity was recorded for 60 min prior to returning them to their home cages.

**Balance beam test:** Balance was assessed by measuring the ability of mice to traverse a narrow beam as described [33,34], with brief modifications. The beam consisted of an 85-cm-long wooden prism, divided into 5-cm frames, with a 1-cm face, placed 40 cm above the bench surface. During the training session, mice were allowed to walk on the beam for 2 min. After 4 h, mice were returned to the beam, and their latency to cover 30 frames and total distance traveled were measured.

**Vertical pole test:** Motor coordination and balance were assessed by measuring the ability of mice to turn and descend from a narrow pole, as described in [33]. The pole consisted of a 60-cm wooden cylinder (1-cm diameter) wrapped in tape to facilitate walking. Mice were trained for 2 consecutive days and tested on the third day. Mice were placed just below the top of the pole facing upwards. Time to completely orient the body downward (time to turn) and time to climb down (time to descend) the pole were measured. An average of three test trials is shown.

**Haloperidol-induced catalepsy:** Mice were intraperitoneally injected with 1 mg/kg Haloperidol (Sigma Aldrich, H-030) or saline vehicle (0.9% NaCl, Teknov, Hollister, CA, USA, S5824). After 30 min, mice were gently positioned in catalepsy position, placing their forelimbs on a 0.5 cm diameter steel rod, covered with non-slippery tape, that was 5 cm above the surface of the bench. A researcher measured the time to remove both front paws from the bar (catalepsy time). Catalepsy was measured every 30 min after the first trial.

**Elevated plus maze:** Anxiety-related behavior was tested by an elevated plus-maze as described in [35].

**Differentiation of neural stem cells (NSCs):** NSCs were generated from induced pluripotent stem cells (iPSCs) as described in [36]. Collagenase detachment media (Type IV, ThermoFisher Scientific, 17104019, 1 mg/mL) in Gibco KnockOut DMEM/F-12 medium (ThermoFisher Scientific, 12660012) was used for iPSC colonies. The cells were transferred to a 0.1% agarose (Sigma-Aldrich, A9414)-coated low-attachment petri dish. The culture dish contained embryonic stem (ES) culture medium Gibco KnockOut DMEM/F12,20% Gibco KnockOut Serum Replacement (ThermoFisher Scientific, 10828028), 100 U/mL penicillin-streptomycin (ThermoFisher Scientific, 15140122), 2.5 mM L-glutamine (ThermoFisher Scientific, 25030081), 1X Non-Essential Amino Acids (ThermoFisher Scientific, 11140050), 15 mM HEPES (ThermoFisher Scientific, 15630106), and 0.1 mM β-mercaptoethanol (ThermoFisher Scientific, 31350010). Embryoid body (EB) differentiation medium [DMEM (Corning, 10-013-CV) supplemented with 20% FBS (ThermoFisher Scientific, 16000036), 1X Non-Essential Amino Acids, 2 mM L-glutamine, and 100 U/mL penicillin-streptomycin] was used every two days replacing 25% ES medium. At day 10, the EBs were attached to dishes coated with poly-L-ornithine (1:1000 in PBS; Sigma-Aldrich, P3655) and laminin (1:100 in KnockOut DMEM/F-12; Sigma-Aldrich, L2020). The EB were cultured in neural induction medium [DMEM/F12,1X N2 (ThermoFisher Scientific, 17502001), 100 U/mL penicillin-streptomycin, 25 ng/mL βFGF (Peprotech, 100-18B), and 25 ng/mL Activin A (Peprotech, Cranbury, NJ, USA, 120-14P). Every 2 days medium was changed. Rosettes were harvested after 7–10 days with the addition of 25 ng/mL Activin A, as described in [37].

**Differentiation into MSNs:** MSNs were prepared as described in [38]. Briefly, 96-well plates were coated with a 50 µg/mL solution of Matrigel (Corning, Corning, NY, USA, CB-40234) for 24 h. Passage 13 NSCs were plated in NPM medium at a concentration of 90,000 cells per well. To start differentiation, NPM medium was replaced with Synaptojuice A. Half-medium changes were done every other day for 7 days. On day eight of differentiation, Synaptojuice A was replaced with Synaptojuice B for 10 days with half-medium changes every other day. Both Synaptojuice A and Synaptojuice B were supplemented with 25 ng/mL of Activin A (Peprotech, 120-14P).

**BCL11B and DARPP-32 immunostaining in MSNs:** Cells were washed with PBS and fixed with 4% paraformaldehyde for 12 min at room temperature. Cells were incubated in block buffer containing 1% normal donkey serum and 0.1% Triton-X-100 in PBS for 1 h. Primary antibodies were diluted at 1:100 in blocking buffer, and the cells were incubated with it overnight. The cells were then washed with buffer containing 0.1% Triton-X-100 in PBS for 5 min, three times. Secondary antibodies were diluted at 1:500 in blocking buffer with 300 nM DAPI, and the cells were incubated in it for 2 h. The cells were then washed three times with 0.1% Triton-X-100 in PBS for 5 min and imaged using a Cytation 5 instrument (Biotek). The antibodies used were rabbit anti-BCL11B (Novus Biological Littleton, CO, USA, NB100-79809) paired with donkey-anti-rabbit Alexa-488 (ThermoFisher, A-21206), and mouse anti-DARPP-32 antibody (Santa Cruz Biotechnology, Inc., Santa Cruz, CA, USA, sc-271111) paired with donkey-anti-mouse Alexa-647 (ThermoFisher, A-31571).

**iPSC-derived MSN qPCR:** MSNs were differentiated in a six-well plate, as described before. RNA was extracted utilizing an ISOLATE II RNA extraction kit (BIO-52071, Bioline). Following the manufacturer’s instructions, 850 ng of RNA per sample were used for cDNA synthesis (Cat No. BIO-65053, Bioline). qPCR Assays for SLIT3 (Cat No. 4453320, ThermoFisher Scientific, Assay ID: Hs00935843_m1), KCNC3 (Cat No. 4448892, ThermoFisher Scientific, Assay ID: Hs01085817_m1), and WNT10A (Cat No. 4448892, ThermoFisher Scientific, Assay ID: Hs05042697_s1) were used. A reaction mix used 1.5 μL of the cDNA template, 5 μL 2x SensiFAST Probe mix (BIO-86005, Bioline), 0.5 μL of ACTB endogenous control (4325788, ThermoFisher Scientific), and 2 μL of molecular grade water (AM9937, Invitrogen). Three technical replicates were done for each sample, and the reactions were run using a Roche LightCycler 480 II.

**Confocal microscopy of nuclear aggregates of BCL11B:** MSNs grown in plastic-bottomed microplates immunostained for BCL11B were imaged using a Zeiss LSM980/Airyscan2 laser scanning confocal microscope, using an LD LCI Plan-Apochromat 40×/1.2 Imm Korr objective lens with glycerol immersion, and Airyscan super resolution mode (41nm/pixel resolution). Images were analyzed in Image Analyst MKII (Version 4.1.3, Image Analyst Software, Novato, CA, USA) using the “Nuclear foci area measurement” standard pipeline, providing counts of BCL11B foci per nucleus, and the area of each nucleus based on the DAPI staining.

## 3. Results

**Figure 1:**
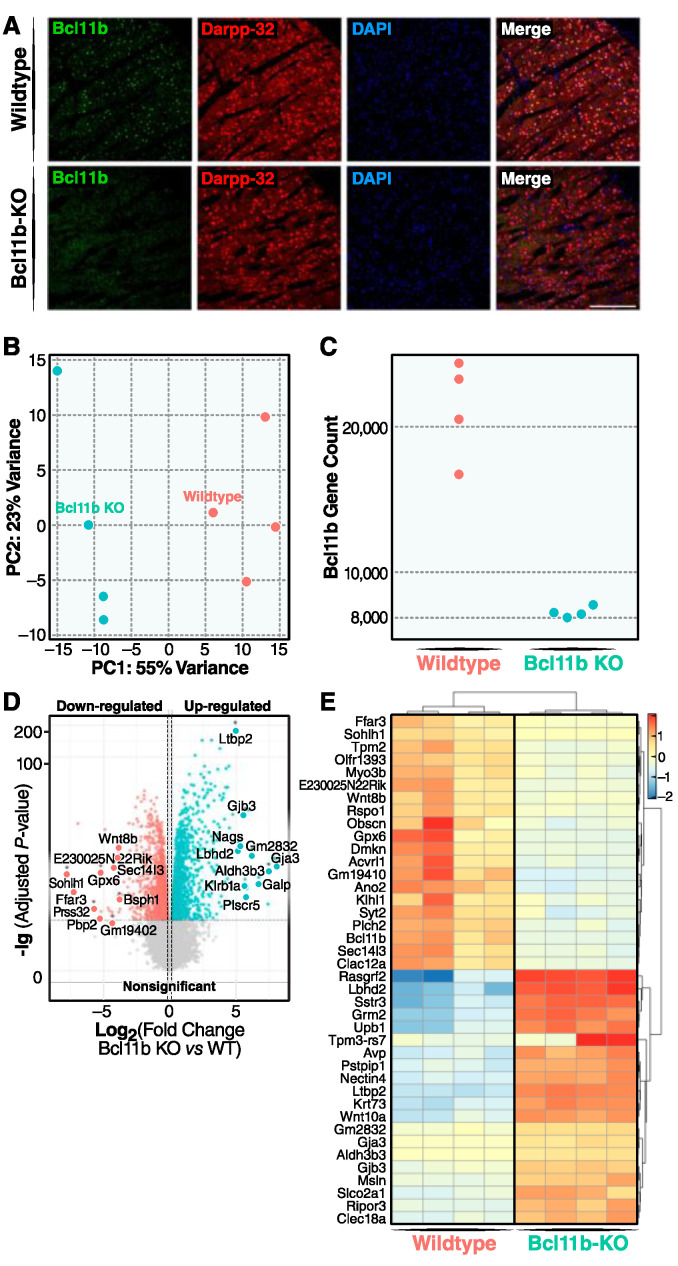
**Identification of transcriptome portraits of *Bcl11b* deletion and WT cells by RNA-seq analysis in Cre+ and Cre− MSNs populations from the striata of D9-Cre mice.** (**A**) *D9-Cre-Bcl11b^tm1.1Leid^* mice specifically knocked down expression *Bcl11b* in MSNs by using a D9-*Cre* under the control of regulatory elements of the mouse Ppp1r1b gene-encoding DARPP-32. *D9-Cre-Bcl11b^tm1.1Leid^* and *Cre*-negative mice (4-months-old) were analyzed using immunohistochemistry (IHC) with a BCL11B antibody. Scale bar is 500 mm. (**B**) PCA plots using the rlog-transformed values indicate a significant difference in the transcriptome *Bcl11b* deleting MSNs, and controls. (**C**) Scatter plot shows that *Bcl11b* gene expression is much less in *D9-Cre-Bcl11b^tm1.1Leid^* mice than *Cre*– control mice. (**D**) Volcano plot shows differences in *Cre*+ and *Cre*− gene expression. Genes with an adjusted *p*-value below 0.01 with absolute log2 fold ratio greater than 1 are highlighted. Genes in red are relatively decreased in expression in the Cre− population (i.e., enriched in the WT population), those in green are relatively increased in expression in the *Bcl11b^tm1.1Leid^* mice, and those in grey are equally distributed among the two populations. (**E**) Heatmap of relative normalized count values across samples. Top 20 up- and downregulated genes that have the highest product of log fold-change and base mean are reported, respectively. Top downregulated genes include: *Bcl11b*, free fatty acid receptor (*Ffar3*), spermatogenesis and oogenesis-specific basic helix-loop-helix 1 (*Sohlh1*), beta tropomyosin (*Tmp2*), *myosin IIIB* (*Myo3b*), wnt family member 8B (*Wnt8b*), R-spondin-1 (*Rspo1*), Obscurin (*Obscn*), glutathione peroxidase 6 (*Gpx6*), dermokine (*Dmkn*), serine/threonine-protein kinase receptor, R3, activin A receptor-like type 1 (*Acvrl1*), anoctamin-2 (*Ano2*), kelch-like protein 1 (*Klhl1*), synaptotagmin-2 (*Syt2*), 1-phosphatidylinositol 4,5-bisphosphate phosphodiesterase eta-2 (*Plch2*), Sec14l3 (uncharacterized protein), and C-type lectin domain family 12 member A (Clec12a).

**Transcriptomic analysis of *D9-Cre*-*Bcl11b*^tm1.1Leid^ mice:** We deleted *Bcl11b* selectively in post-mitotic, post-migrational adult MSNs by using a Cre mouse with the regulatory components of the genomic elements of DARPP-32 (i.e., D9) [30]. D9-Cre mice were crossed with *Bcl11b^tm1.1Leid^* loxP-flanked mutant mice. Using immunostaining, we confirmed that *D9-Cre-Bcl11b^tm1.1Leid^* mice express almost no Bcl11b in the MSNs at 5 weeks of age (Figure 1A). We performed RNA-sequencing on striatal tissue from homozygote-floxed *Cre^−^* and *D9-Cre-Bcl11b^tm1.1Leid^* mice at 4 months of age. We detected 38,386 mRNAs, including hundreds of long-intergenic non-coding RNAs (lincRNA), and 938 miRNAs. A differential expression analysis revealed that the deletion of *Bcl11b* resulted in 2771 differentially expressed genes (DEGs) with an adjusted *p*-value of less than 0.01 (Table S1). Among these DEGs, 1536 were upregulated and 1235 were downregulated (Table S1). The PCA plot shows the separation in the clustering of the transcriptome of the *Cre^−^* and *D9-Cre-Bcl11b^tm1.1Leid^* mice (Figure 1B). As expected, the levels of *Bcl11b* were substantially reduced in the *D9-Cre-Bcl11b^tm1.1Leid^* mice (Figure 1C). The volcano plot and heatmap highlight the top enriched genes in the *D9-Cre*-*Bcl11b^tm1.1Leid^* mice (Figure 1D,E). Although the top downregulated genes were not specifically correlated with MSN function, importantly, *Bcl11b* deletion also resulted in the loss of MSN-enriched markers with a reduced expression of: forkhead box protein P1 (*FoxP1*), DARPP-32 (also known as *Ppp1r1b*); *Arpp21*, proenkephalin-A (*Penk*), 5-hydrotryptamine receptor 1B,1D (*Htr1b, Htr1D*), ryanodine receptor (*Ryr1*), GABA receptor subunit delta; alpha-4 (*Gabrd, Gabra4*), histamine H3 receptor (*Hrh3*), *Drd1*, *Drd2*, and metabotropic glutamate receptor 1 (*Grm1*) (Table S1). Notably, *Drd2* (log2fold = −0.60) was reduced more than *Drd1* (log2fold = −0.26). Both striosome and matrix genes were altered.

Although many markers specifically associated with MSNs were downregulated, there were some notable exceptions (Figure 1D,E). One of the top upregulated genes was latent transforming factor beta binding protein (*Ltbp2*). It is critical in the TGFβ signaling and regulation of this pathway that it has been linked to MSN developmental processes and HD neuropathogenesis [1]. Correspondingly, activin A receptor like type 1 (*Acvrl1)*, is upregulated. During the development of the lateral ganglionic eminence (LGE), the ligand for Acvr1, Activin A, plays a critical role in the specification of striatal fate [39,40], and both activin receptors and activated activin are expressed in the developing LGE [39,40]. *Wnt8b* is involved in the caudalization of a regional identity [41]. Upon the reduction of *Bcl11b*, ras-specific guanine-nucleotide releasing factor 2 (*RasGRF2*) was also reduced. This calcium-regulated exchange factor [42] alters the ERK-dependent cocaine reward in mice [43]. Some of the top upregulated genes are involved in cell death signaling pathways (e.g., *Clec12a*, a uric acid receptor that potentiates type I interferon responses) [44]. *Sstr3* is a G-protein-coupled receptor (GPCR) whose signaling affects neuronal cilia and apoptosis and is upregulated after heroin exposure [45,46]. In addition, glutamate metabotropic receptor 2 (*Grm2*) is increased in the *Bcl11b* MSN-deletion mice. A group of top dysregulated genes are not known to be specifically associated with MSNs and therefore warrant further investigation (Figure 1D,E, Table S1).

We conclude that the reduction in *Bcl11b* affects multiple genes involved in MSN maintenance and identity and signal transduction.

**Figure 2:**
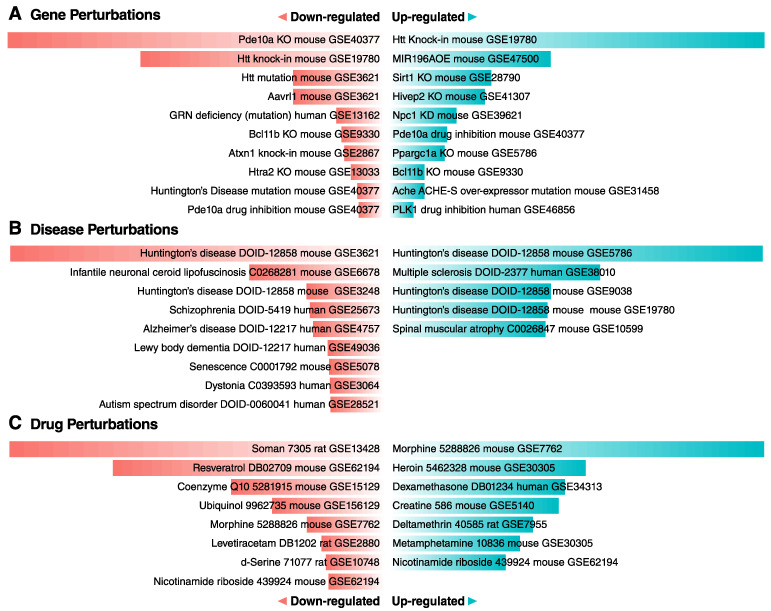
**Transcriptional profile of *Bcl11b* deletion is highly correlated with HD mouse models and postmortem human HD tissue.** (**A**) Gene perturbation enrichment analysis. (**B**) Disease perturbation enrichment analysis. (**C**) Drug perturbation enrichment analysis.

***Bcl11b* reduction results in differentially expressed genes that correlate with pathways dysregulated in HD.** We used Enrichr [47] to identify the co-expression networks that most overlap with the transcriptomics of the Bcl11b reduction in MSNs (Figure 2). Strikingly, the expression changes for the *D9-Cre-Bcl11b^tm1.1Leid^* mice overlapped with expression profiles of HD mouse models, postmortem HD tissue and/or knockout mice (KO), including the genes *Pde10a*, *Sirt1*, *Htra2*, *Npc1,* and *Ppargc1a* (Figure 2A,B). Many of the overlapping genes are markers of MSNs, and the loss of striatal MSN identity overlaps with the *D9-Cre-Bcl11b^tm1.1Leid^* transcriptomics. The *D9-Cre-Bcl11b^tm1.1Leid^* mice transcriptional profile had drug perturbations from the GEO database that overlapped with soman, morphine, resveratrol, heroin, dexamethasone, coenzyme Q, creatine, levetiracetam, methamphetamine, and nicotinamide riboside (Figure 2C). These drug perturbations correlate with striatal function or known drug targets in HD. We also evaluated the overlap of the gene expression profiles of the 10-month-old Q175 knockin mouse model [21] and the conditional *D9-Cre-Bcl11b^tm1.1Leid^* mice. There were 683 genes shared when comparing the transcriptomic data sets (zQ175, 2795 genes) with a *p*-value of 3.75E-76, when using the Fisher exact test. The top KEGG pathway (2021 human) is the dopaminergic synapse, and the protein-protein interaction hub protein is GRIN1. The shared genes have ontologies for the regulation of neurotransmitter receptor activity, calcium signaling, potassium channel regulation, protein/threonine kinase activity, activin receptor activity, glutamate receptor activity, and postsynaptic density. Kinase regulation includes CAMK4 and the regulation of a glutamate receptor by CK1 and CDK5. As expected, our data enriches to constitutively deleted Bcl11b mice. Table S2 summarizes the overlap with the majority of known mouse HD transcriptomics data sets and the overlap with the *D9-Cre-Bcl11b^tm1.1Leid^* mice.

The top canonical pathways identified by a complementary analysis with an Ingenuity Pathway Analysis (IPA) were the opioid signaling pathway (*p*-value 6.22E-14), cAMP-mediated signaling (*p*-value 3.60E-08) via which dopamine signals are transduced, the synaptogenesis signaling pathway (*p*-value 4.07E-8), protein kinase A signaling (*p*-value 4.11 × 10^−8^), and calcium signaling (*p*-value 1.10E-07). The top upstream regulators were levodopa, CREB1, amino-5-phosphonovaleric acid, and huntingtin (HTT).

**Figure 3:**
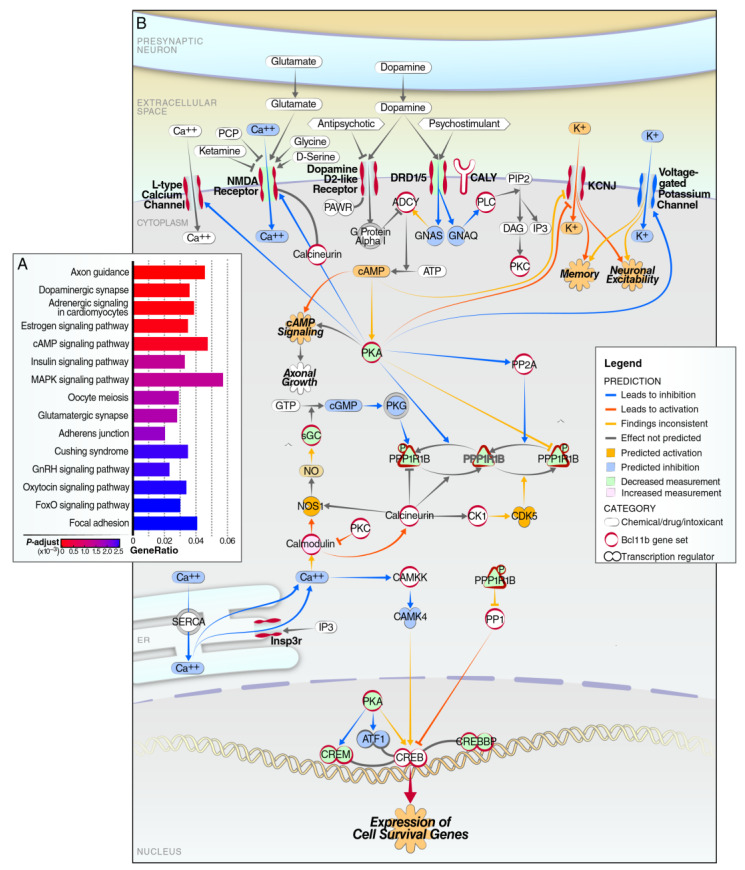
**Significantly enriched KEGG terms and IPA signaling.** (**A**) KEGG term enrichment analysis of gene signatures altered by *Bcl11b* deletion highlighted the axon guidance, dopaminergic synapses, adrenergic, estrogen, cAMP, MAPK, insulin, oocytes, and glutamatergic signaling. (**B**) IPA signaling pathway highlights for dopamine DARPP-32 feedback cAMP signaling.

Next, we carried out a term enrichment analysis for GO or KEGG processes or functions associated with the DEGs for the conditional *D9-Cre-Bcl11b^tm1.1Leid^* mice, compared to the controls (Figure 3, Table S1, Supplemental Figure S1). The KEGG term enrichment analysis for gene signatures altered by *D9-Cre-Bcl11b^tm1.1Leid^* highlighted the axon guidance, dopaminergic synapses, adrenergic, estrogen, cAMP, MAPK, insulin, oocytes, and glutamatergic signaling (Figure 3A). An IPA analysis summarizes the critical pathway for dopamine DARPP-32 feedback cAMP signaling that is enriched in the *D9-Cre-Bcl11b^tm1.1Leid^* mice (Figure 3B). This is a key pathway disrupted in HD. Interestingly, many of the genes with a log fold-change >1.0 correlated with genes involved in calcium homeostasis (Table S1, Supplemental Figure S2).

Enriched BPs included synapse organization, functions, or molecules related with transmembrane activities, such as a transmembrane transporter or ion channels (Supplemental Figure S1A). Correspondingly, the GO terms enriched for BPs in the downregulated genes were protein localization, dephosphorylation, the rhythmic process, and the postsynapse. The cellular components and molecular functions are also shown in Supplemental Figure S1B–G. Top upstream terms and network from the IPA analysis were HTT, NR4A1, CNTF, epilepsy, dyskinesia, synaptic depression, the organization of cells, and catalepsy (Supplemental Figure S3). The identification of NR4A1 as a top upstream regulator is interesting. Although *Bcl11b* appeared to regulate gene expression in both striosomes and in the matrix in our current study, *Nr4a1* is highly enriched in the striosomes and is required for their development [3,31], and striosomes are altered in HD [48,49,50].

Thus, a decrease in *Bcl11b* alters the general neuronal and MSN-specific processes, including in synapse organization and functions, or in molecules related with transmembrane activities (e.g., transmembrane transporter and ion channels).

**Figure 4:**
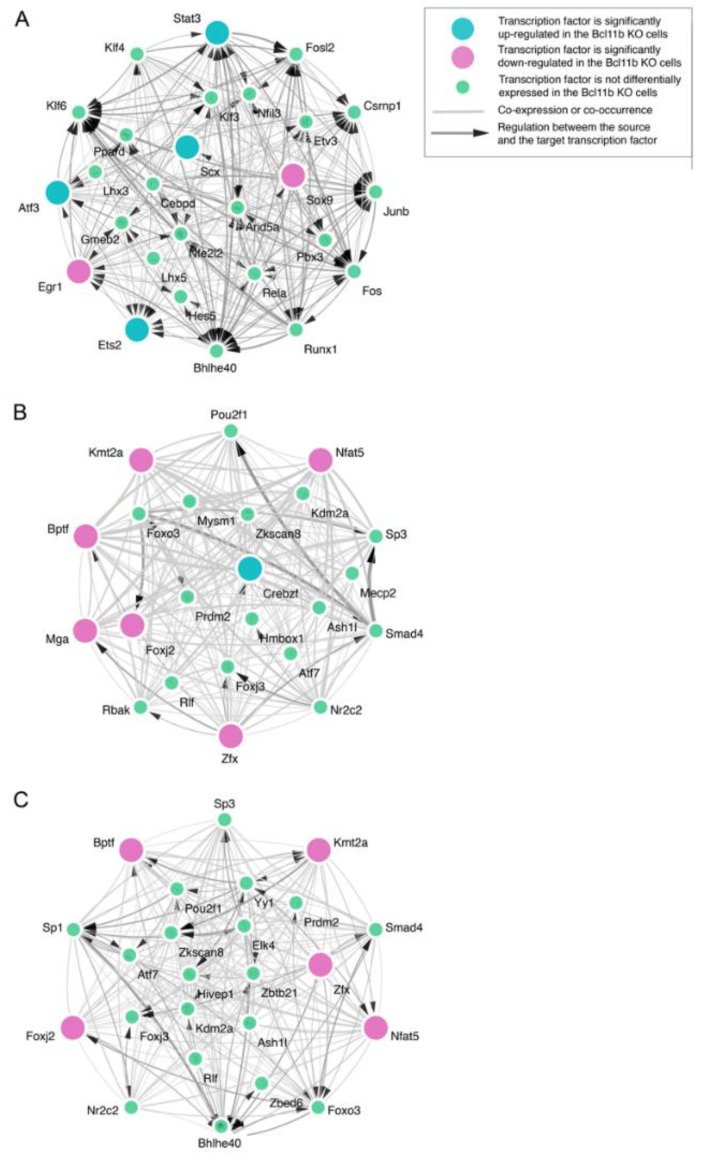
**Gene regulatory network analysis reveals critical up-stream TFs from the gene signatures altered by *Bcl11b* deletion.** Gene regulatory network analysis. (**A**) Differentially expressed TFs that are upregulated (**A**) or downregulated (**B**) in *Bcl11b*-deletion cells. (**C**) All differentially expressed TFs in *Bcl11b*-deletion cells.

**Transcriptional network impacted by *Bcl11b* deletion:** We evaluated the TFs and networks impacted by the deletion of *Bcl11b*. Using the mouse TF database, a total of 287 differentially expressed TFs were altered [51]. Among these, 109 were upregulated, and 178 were downregulated (Table S1). These differentially expressed TFs were used as the input for the gene regulatory network analysis to determine the key upstream regulators in the *D9-Cre-Bcl11b^tm1.1Leid^* MSNs (Figure 4). From the inferred networks, we identified the hub gene, *Egr1* (Figure 4A), which plays a key role in the induction of DARPP-32 expression in MSNs [52]. The enrichment of *Foxo3*, *Foxj2*, and *Foxj3* showed that the decrease of *Bcl11b* alters the forkhead pathway (Figure 4B), which is important in adult human neurogenesis and cell-cycle inhibition [53,54].

Additionally, our TF network identifies *Kmt2a*, *Kdm2a*, and *Ash1l*, which are important histone-modifying enzymes involved in chromatin remodeling (Figure 4B,C). Kmt2a modifies H3K4 [55], Ash1l modifies H3K36 [56], and Kdm2a modifies H3K4 and H3K36 [57,58], and these may indicate that a deficiency in *Bcl11b* causes abnormal histone modification. Notably, H3 lysine 4 trimethylation (H3K4me3) at transcriptionally repressed promoters in the brain is considered an early feature of HD [59].

Other noteworthy TFs that were enriched in the networks were *Nfat5* and *Nfatc3*. They regulate the calcineurin-mediated signaling pathway, and importantly, calcineurin inactivates DARPP-32 by dephosphorylation [60]. Moreover, hyperactivated calcineurin dysregulates BDNF transport in HD [61,62].

Overall, *D9-Cre-Bcl11b^tm1.1Leid^* mice strongly mimic aspects of HD transcriptional dysregulation.

**Figure 5:**
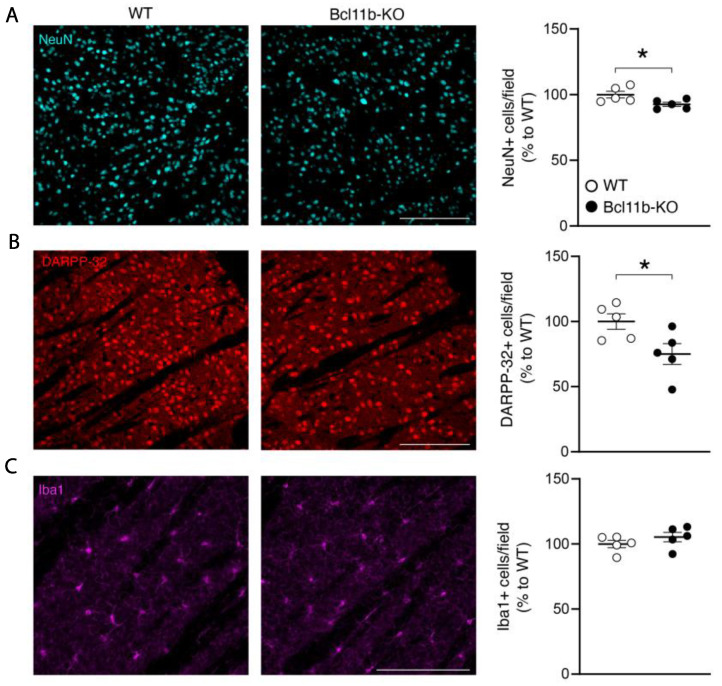
***Bcl11b* deficiency leads to a reduced number of MSNs without microgliosis.** Representative striatal images immunostained with cell type-specific markers, and graphs detailing corresponding quantification. (**A**). IHC of *Bcl11b^tm1.1Leid^* and control mice IHC immunostained with NeuN (neurons) and quantification. (**B**)**.** IHC of *Bcl11b^tm1.1Leid^* and control mice immunostained with DARPP-32 (MSNs) and quantification. (**C**)**.** IHC of *Bcl11b^tm1.1Leid^* and control mice with Iba1 (microglia). Scale bars, 200 mm. Graphs show the number of NeuN, DARPP-32, and Iba1-positive cells in the striatum. Each point represents an individual mouse. All data are shown as mean  ±  SEM (WT *n*  =  5; *Bcl11b*-KO *n* = 5.) Two-tailed unpaired t-test, * *p* < 0.05.

The decrease of *Bcl11b* in differentiated MSNs resulted in a decrease in NeuN+/DARPP-32+ cells, motor deficits, and a decreased response to haloperidol. Selective loss of striatal MSNs is a major hallmark in HD but is poorly recapitulated in mouse models [63]. To determine if *Bcl11b* deletion compromises neuronal viability, we counted the striatal neurons, and specifically MSNs, using a NeuN and DARPP-32 immunofluorescence. Fewer NeuN+ and DARPP-32+ cells were detected in the striatum of *D9-Cre-Bcl11b^tm1.1Leid^* mice than in the wildtype (WT) mice (Figure 5A,B). The decreased neuronal numbers were not accompanied by increased numbers of microglia, as revealed by Iba1 immunostaining (Figure 5C). The decrease in NeuN+ and DARPP-32+ cells may suggest a loss of neurons, de-differentiation, or a lack of differentiation.

**Figure 6:**
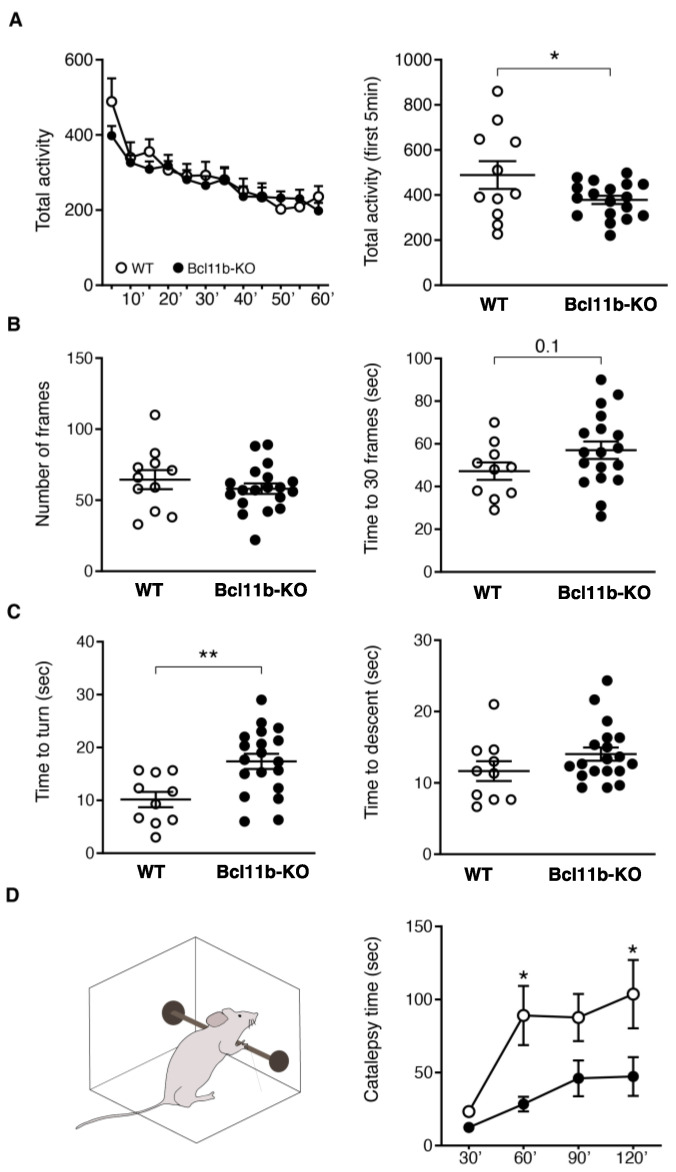
***Bcl11b* deficiency in adult mice partly recapitulate HD-associated motor phenotype.** (**A**). Spontaneous locomotor activity measured in the *Bcl11b* deficiency mice. (**B**). Balance beam: from *left* to *right*; numbers of frames crossed in 2 min and times to cross 30 frames. (**C**). Vertical pole: times to turn (*left*) and times to descend (*right*) were recorded after placing the mice upwards to the pole. Three trials were conducted, and data represent the mean ± SEM (WT *n* = 11, *D9-Cre-Bcl11b^tm1.1Leid^* mice *n* = 18). Two-tailed unpaired *t*-test, * *p* < 0.05; ** *p* < 0.01. (**D**). Schematic diagram of catalepsy position (left). Catalepsy time after Haloperidol treatment (right). Two-Way ANOVA, with Bonferroni as post-hoc test * *p* < 0.05.

The role of the striatum in movement and the overlap of the gene expression changes with HD prompted us to evaluate the motor behavior of striatal *D9-Cre-Bcl11b^tm1.1Leid^* mice. We found that spontaneous locomotor activity was reduced in *D9-Cre-Bcl11b^tm1.1Leid^* mice in the first 5 min (Figure 6A). There was a trend towards subtle balance alterations in the balance beam test, in that *D9-Cre-Bcl11b^tm1.1Leid^* mice and WT mice crossed a similar number of frames, but the KO mice appeared to require more time to cross 30 frames than WT mice (*p*-value = 0.096; *t*-test) (Figure 6B). Importantly, *D9-Cre-Bcl11b^tm1.1Leid^* mice displayed poor performance in the vertical pole test, requiring more time to turn and descend than WT mice (unpaired *t*-test, * *p* < 0.05; ** *p* < 0.01) (Figure 6C). *Bcl11b* deletion did not alter anxiety-like behavior in the elevated plus maze (Supplemental Figure S4). These results suggest that motor abnormalities after *Bcl11b* deletion in adult MSNs overlap to some extent with HD mouse models.

Next, RNA-seq analysis pointed to specific alterations of genes involved in the dopaminergic synapse pathway (adjusted *p*-value = 3.27E10−7). A cell-type-specific enrichment analysis showed that *Bcl11b* deletion caused a downregulation of genes that were enriched in both D1 and D2-MSN subtypes (Supplemental Figure S4). Notably, *Drd2* (log2fold = −0.60) was reduced more than *Drd1* (log2fold = −0.26). To functionally explore D2R-mediated behavior, we performed the haloperidol-induced catalepsy test. Haloperidol treatment in mice produces a behavioral state in which the mice fail to correct externally imposed postures (i.e., catalepsy). Integrity of postsynaptic dopamine receptors is required to observe this phenotype [64]. Haloperidol (1 mg/kg) was injected intraperitoneally into WT and D9-Cre *Bcl11b*-deletion mice. Catalepsy was measured 30 min after the injection and every 30 min up to 2 h. Catalepsy time was lower in *Bcl11b*-deletion mice than WT littermates (Figure 6D, two-way ANOVA, with Bonferroni post-hoc test, * *p* < 0.05).

**Figure 7:**
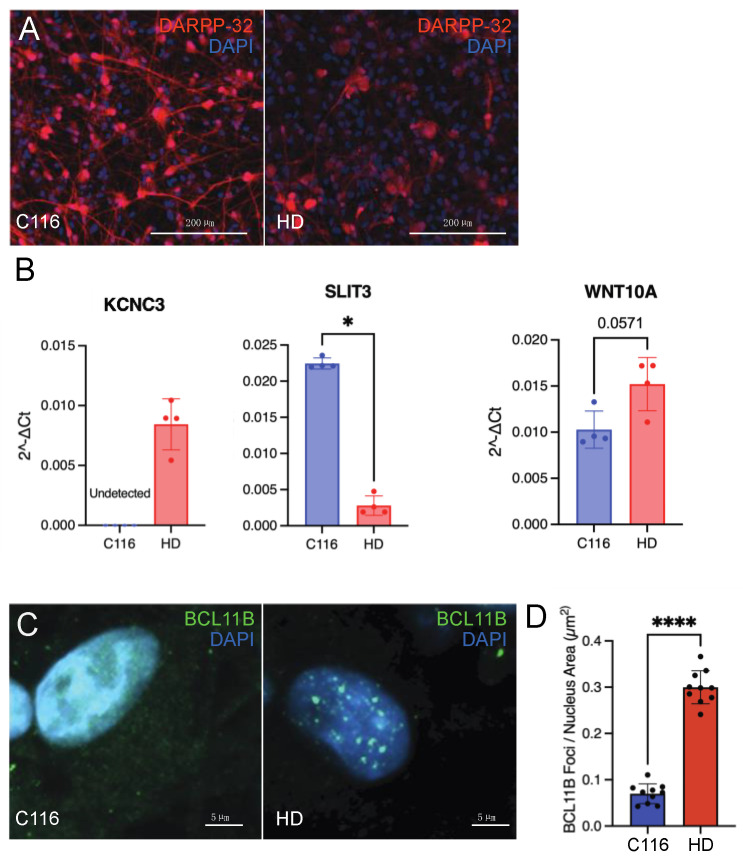
**Human MSNs derived from HD patient iPSCs reveals mislocalization of BCL11B into nuclear aggregates.** (**A**). Isogenic HD72 and C116 MSNs differentiated from iPSCs immunostained with DARPP-32. (**B**). Top genes dysregulated in *D9-Cre-Bcl11b^tm1.1Leid^* mice follow similar trends in expression as measured by RT-PCR. KCNC3 and WNT10A are upregulated in HD72-MSNs, compared to control C116. Like the *D9-Cre-Bcl11b^tm1.1Leid^* mice transcriptomics, SLIT3 was downregulated in HD-MSNs. (**C**). IHC with BCL11B antibody show more large nuclear aggregates in the HD72-MSNs than in C116-MSNs. (**D**). Quantification of the BCL11B foci per nuclear area in isogenic HD72 and C116-MSNs. * *p* < 0.05; **** *p* < 0.0001, Mann Whitney test.

**Disruption of BCL11B function in a human HD MSN model.** The strong correlation of the *D9-Cre-Bcl11b^tm1.1Leid^* mice transcriptome with HD models prompted us to determine how the *HTT* mutation mimics lower levels of *Bcl11b*. We differentiated isogenic human patient HD72 iPSCs (CAG repeat size 72) into MSNs (Figure 7A). As expected, the HD72-MSNs had lower levels of DARPP-32 than isogenic control C116-MSNs. Top genes dysregulated in *D9-Cre-Bcl11b^tm1.1Leid^* mice follow similar trends in expression as measured by RT-PCR in human HD72-MSNs (Figure 7B). KCNC3 and WNT10A were *upregulated* in HD72-MSNs, compared to control C116. Like the *D9-Cre-Bcl11b^tm1.1Leid^* mice transcriptomics, SLIT3 was downregulated in HD72-MSNs. BCL11B was modestly upregulated but not statistically significant (data not shown), and may represent the fact that the iPSC-derived MSNs are relatively immature, compared to mouse adult MSNs in vivo. As our current studies show a loss of *Bcl11b* in MSNs correlates with the HD transcriptome, we investigated the mechanism for how this might occur in HD. BCL11B expression was characterized by immunofluorescence in HD72-MSNs compared to control. In HD72-MSNs, BCL11B was concentrated in internuclear aggregates, but showed a diffuse pattern in C116-MSNs (Figure 7C). Many more internuclear aggregates were noted in the HD72-MSNs than in controls (Figure 7D). We conclude that the sequestration of BCL11B into nuclear aggregates may lead to loss of transcriptional activity of BCL11B in HD even in the presence of normal level of expression.

## 4. Discussion

We report that selective Cre-mediated deletion of the transcription factor *Blc11b*/*Ctip2* in differentiated striatal MSNs leads to a transcriptional signature similar to HD and supports the notion that *Bcl11b* has a critical role in maintaining key pathways in the biological function and identity of MSNs in adult mice. Reduction in *Bcl11b* results in the lower expression of MSN differentiation markers, including *FoxP1*, *DARPP-32* (*also known as Ppp1r1b*), *Arpp21*, *Penk*, *Htr1b*, *Htr1D*, *Ryr1*, *Gabrd*, *Gabra4*, *Hrh3*, *Drd1*, *Drd2* and *Grm1*. Previous studies have shown that loss of *Bcl11b* during development results in deficits in MSN birth, migration and differentiation [15]. Our results, therefore, are consistent with a continued role of *Bcl11b* in MSN differentiation and/or maintenance of identity in adult mice.

A cell-type-specific enrichment analysis showed *Bcl11b* deletion caused a downregulation of genes that were enriched in both D1 and D2-MSN subtypes. Notably, *Drd2* was reduced more than *Drd1*. We also found alterations in MSN gene expression in both the patch/striosome and matrix compartments of the striatum, without enrichment for either compartment. This includes the striosome markers *Oprm1*, *Tac1*, *Spon1*, *Lydp1*, *Kremmen1*, *Tshz1*, and *Pdyn* for patch and the matrix marker, *Epha4*. We functionally validated that the gene expression changes were large enough to compromise dopamine neurotransmission, as evidenced by an abnormal haloperidol-induced catalepsy test.

The expression changes for the *D9-Cre-Bcl11b^tm1.1Leid^* mice overlapped with the gene expression profiles of HD mouse models and the postmortem HD tissue. Interestingly, some gene expression changes that overlapped with *D9-Cre-Bcl11b^tm1.1Leid^* mice are genes involved in HD pathophysiology including *Pde10a*, *Sirt1*, *Htra2*, *Npc1,* and *Ppargc1a*. Transcriptional dysregulation has long been described as an important pathological change in HD. Many of the downregulated genes in HD striatum are enriched for genes that define MSN identity and function [21,65,66,67,68,69,70]. Further, as in HD, genes whose expression are altered after the depletion of *Bcl11b* in MSNs were enriched in calcium and HDAC signaling. The mechanism behind mutant HTT-induced transcriptional effects is unclear. Our studies using human HD-MSNs suggests that the sequestration of BCL11B into nuclear aggregates may lead to loss of function of BCL11B in HD and the loss of MSN identity and function. This is consistent with a physical interaction of BCL11B with mHTT [19] and altered levels in HD mouse models [22].

Our results highlight a cascade of TFs that are impacted when *Bcl11b* is deleted in striatal MSNs. As discussed above, we identified that *Egr1,* required for DARPP-32 expression, is a hub gene [52]. Th enrichment of *Foxo3*, *Foxj2*, and *Foxj3* showed that the deficiency of *Bcl11b* alters the forkhead pathway which is important in adult human neurogenesis and cell-cycle inhibition [53,54]. Stat1/3 are enriched in the differentially expressed TFs that are upregulated in the case of *Bcl11b* deletion MSNs. We recently identified that Stat1/3 is a TF that is required for striosome development [31]. Our results suggest that this may be an important pathway in adult MSNs as well for striosome identity maintenance.

Recent studies have used CRISPR/Cas9 to deplete human embryonic stem cells of *BCL11B*. The reduction of BCL11B in human MSNs leads to neuronal vulnerability and dysfunction. In the human model of MSNs where BCL11B is depleted, cAMP-Ca^2+^ signaling, which integrates the PKA pathway, was identified as dysregulated [20]. These same pathways were identified in the current study. *BCL11B* knockdown likely leads to common alterations in signaling in both mice and human models.

## 5. Conclusions

In conclusion, dissecting the role of *Bcl11b* in adult striatal MSNs has provided valuable information on its function as well as supporting its role in basal ganglia diseases, such as HD. The postnatal deletion of *Bcl11b* in MSNs mimics aspects of the phenotype identified in genetic HD mouse models. However, the *D9-Cre-Bcl11b^tm1.1Leid^* mouse does not constitute an actual model of human HD, and certainly there are many other, multicellular contributions to the HD phenotype. Finally, *Bcl11b* has a critical role in the maintenance of mature MSN phenotype and function, with a very distinct overlap with the HD transcriptome, via which its decrease may contribute to HD pathogenesis.

## Data Availability

The deposited raw data for the transcriptomics are GSE185476.

## Supplementary Materials

The following supporting information can be downloaded at: https://www.mdpi.com/article/10.3390/biomedicines10102377/s1. Figure S1: GO enrichment of MSN Bcl11b deficiency; Figure S2: Calcium signaling pathways enriched in Bcl11b deficiency; Figure S3: IPA analysis of MSN mouse Bcl11b deficiency; Figure S4: Bcl11b deficiency does not induce anxiety-like behaviors. FigureS5: Cell-type enrichment analysis.; Table S1: Transcriptomics of *Bcl11b* deficiency with functional analysis; Table S2:HD mouse transcriptomics overlaps with *Bcl11b* data set.
