# Supplementary material for: Postnatal Conditional Deletion of Bcl11b in Striatal Projection Neurons Mimics the Transcriptional Signature of Huntington’s Disease
Source: Biomedicines. 2022 Sep 23;10(10):2377. doi: 10.3390/biomedicines10102377 (PMC9598565; doi:10.3390/biomedicines10102377)
Supplement: Supplementary file 1 [file biomedicines-10-02377-s001.zip › biomedicines-1828052-supplementary.pdf]

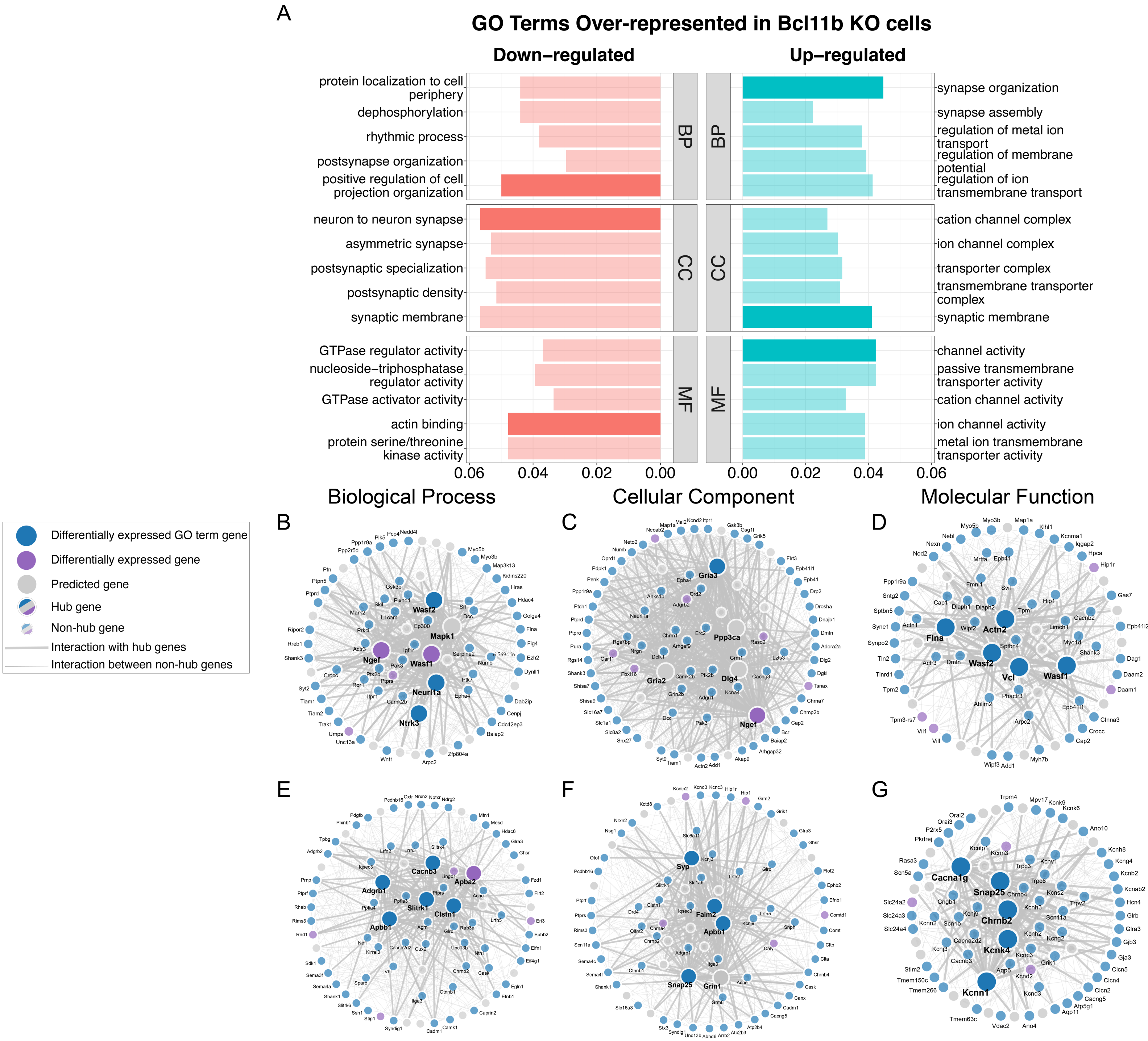

Supplemental Figure S1. GO enrichment of MSN Bcl11b deficiency. A. GO term enrichment analysis on the gene signatures altered by Bcl11b deficiency, with the most significantly enriched GO terms highlighted including positive regulation of cell projection organization, neuron-to-neuron synapse, actin binding, synapse organization, synaptic membrane. Hub gene analysis of the protein-protein interaction network identified in the most significantly enriched GO terms by different categories and expression patterns. B. Positive regulation of cell projection organization C. Neuron-to-neuron synapse. D. Actin binding. E. Synapse organization. F. Synaptic membrane. G. Channel activity.

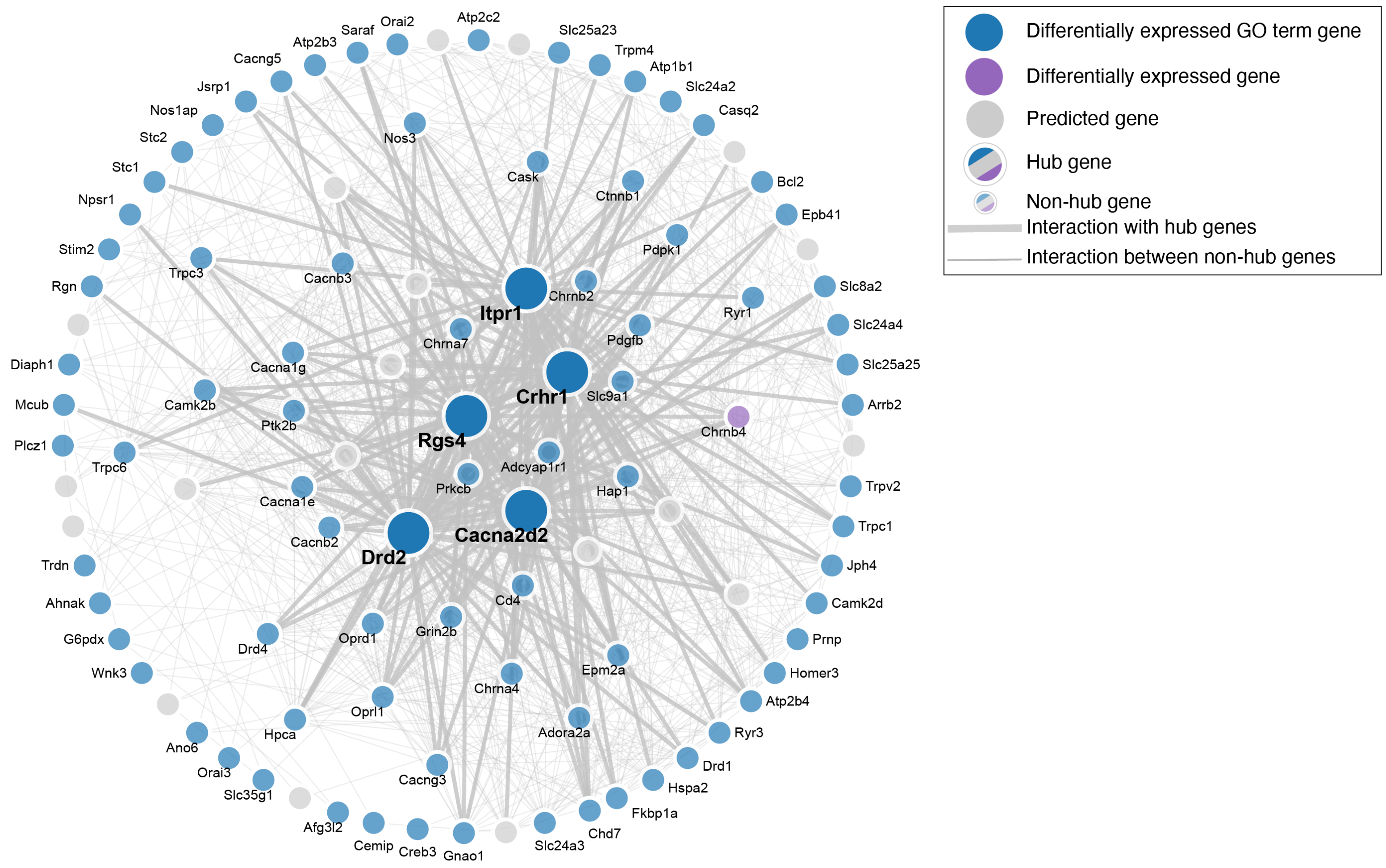

Supplemental Figure S2. Calcium signaling pathways enriched in Bcl11b deficiency.

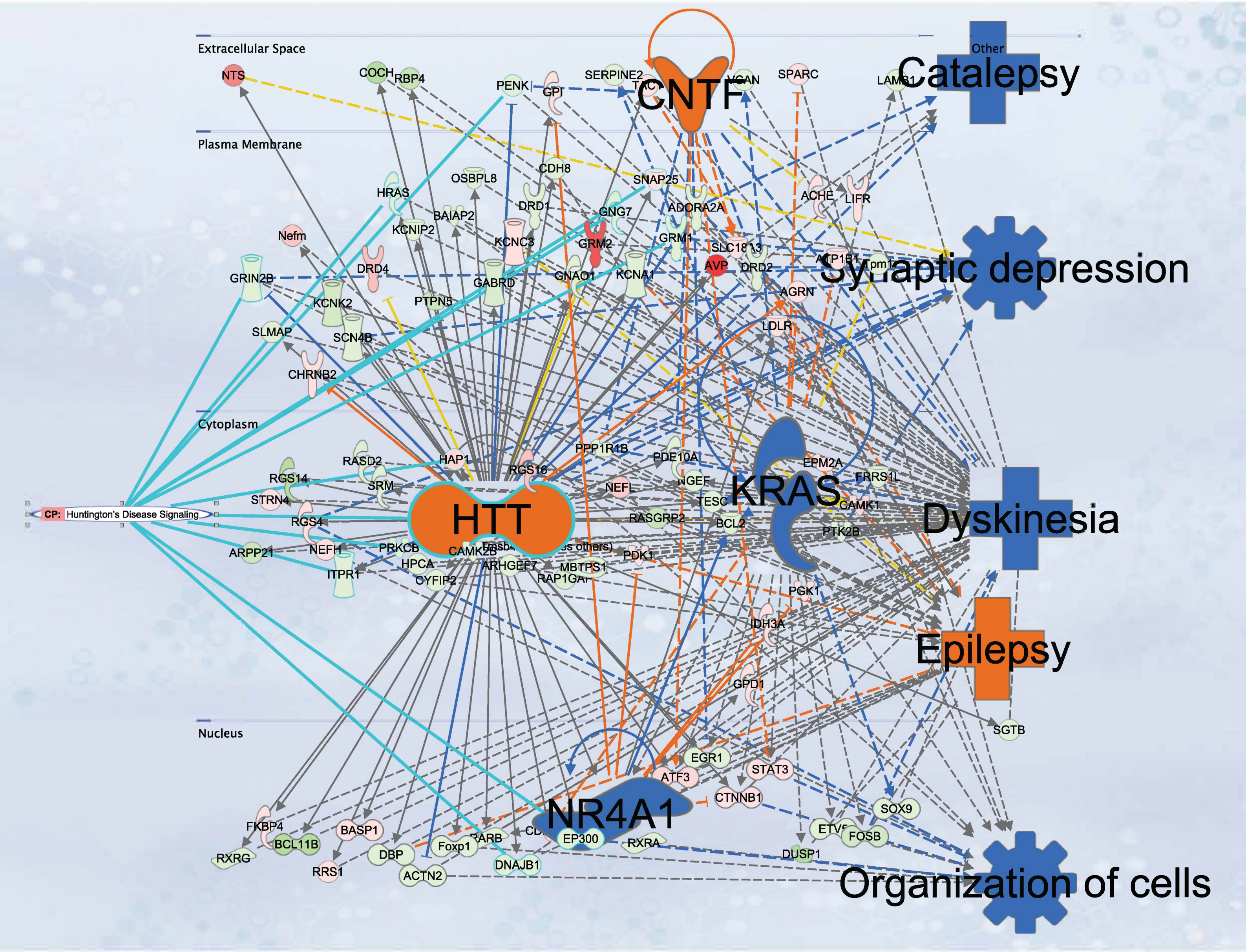

Supplemental Figure S3. IPA analysis of MSN mouse Bcl11b deficiency. Top upstream terms and network from IPA analysis are HTT, NR4A1, CNTF, epilepsy, dyskinesia, synaptic depression, organization of cells and catalepsy. The interconnecting genes are shown in the network.

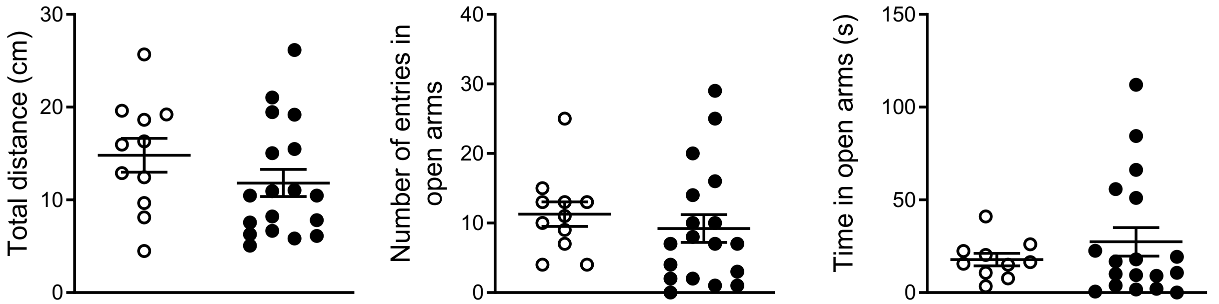

Supplemental Figure S4. Bcl11b deficiency does not induce anxiety-like behaviors. Elevated plus maze. Bcl11b$^{tm1.1Lead}$ mice did not exhibit any differences in the total distance, number of entire sin open arms or time spend in open arms. Data represent the mean ± SEM (WT n = 11, Bcl11b KO n = 18).

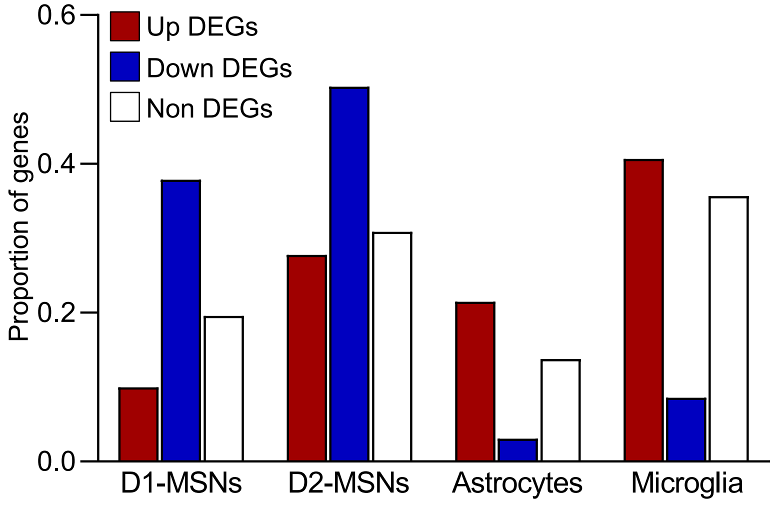

Supplemental Figure S5. Cell-type enrichment analysis. Proportion of up-regulated (red) and down-regulated (blue) differentially expressed genes, and non-differentially expressed genes (white) overlapping with cell-type specific genes.
